# Microsecond Electrical Breakdown in Water: Advances Using Emission Analysis and Cavitation Bubble Theory

**DOI:** 10.3390/molecules27030662

**Published:** 2022-01-20

**Authors:** Cathy Rond, Nicolas Fagnon, Benjamin Dufour, Son Truong Nguyen, Arlette Vega, Xavier Duten

**Affiliations:** Laboratoire des Sciences des Proécédés et des Matériaux LSPM-CNRS UPR3407, Université Sorbonne Paris Nord, 93430 Villetaneuse, France; fagnon@lspm.cnrs.fr (N.F.); dufour@lspm.cnrs.fr (B.D.); nguyen@lspm.cnrs.fr (S.T.N.); vega@lspm.cnrs.fr (A.V.); duten@lspm.cnrs.fr (X.D.)

**Keywords:** electrical discharge, plasma in liquid, emission spectroscopy, cavitation bubble, discharge regimes

## Abstract

Electrical discharges in water are a subject of major interest because of both the wide range of potential applications and the complexity of the processes. This paper aimed to provide significant insights to better understand processes involved during a microsecond electrical discharge in water, especially during the propagation and the breakdown phases. Two different approaches were considered. The first analysis focused on the emission produced by the discharge during the propagation using fast imaging measurements and spatially resolved optical emission spectroscopy. The excited species H, O, and OH were monitored in the whole interelectrode gap. The second analysis concerned the thermodynamic conditions induced by the breakdown of the discharge. The time evolution of the bubble radius was simulated and estimation of the initial pressure of the cavitation bubble was performed using the Rayleigh–Plesset model. Values of about 1.7 × 10^7^ Pa and 1.2 × 10^8^ Pa were reported for the cathode and anode regimes, respectively. This multidisciplinary approach constitutes a new step to obtain an accurate physical and chemical description of pin-to-pin electrical discharges in water.

## 1. Introduction

Since a few decades, electrical discharges in water have represented a highly topical issue due to the wide range of potential applications that have been identified such as nanomaterial synthesis [1,2], medical treatment [3], or environmental applications [4,5]. It has been shown that discharges in water are an efficient tool for the production of active species that play a major role for many processes [6,7]. However, the promising results in terms of applications are limited by the complexity of the chemical and physical phenomena involved. As an example, two theories, bubble theory and direct ionization theory, are still under discussion to explain the formation and propagation mechanisms of plasma in liquids [8,9].

The variability in the results reported by previous investigations attests that the discharge characteristics strongly depend on the experimental setup such as the electrodes’ configuration, the liquid properties, and the power supply. In particular, many different types of propagation modes have been reported and their characteristics have been analyzed [10,11,12,13]. In addition to providing significant steps toward a better comprehension of the mechanisms, these works have shown that the complexity of the discharge in liquid requires but also limits the use of various different diagnostics. The main tools are the refractive index methods for the discharge regime analysis [14,15,16] or emission spectroscopy for the determination of the chemically active species [17,18]. Very few works have reported a global study by coupling these diagnostics. As an example, Grosse et al. studied the ignition, cavitation, and plasma parameters for nanosecond discharges in water [19]. Despite some common features among the different discharge regimes, they presented specificities related to the experimental conditions that need to be specifically analyzed.

This work focused on the pin-to-pin electrode configuration delivering a high-voltage microsecond pulse (500 µs duration) in water of medium conductivity. This system has the particularity to provide different discharge regimes for the same experimental conditions. In previous studies, two main regimes have been identified and present various features for every phase of the discharge: initiation, propagation, breakdown, and post-breakdown [20,21,22]. They have been called cathode and anode regimes in accordance with features observed in classical pin-to-plane configurations. These studies using both schlieren and electrical measurements have initiated reports of the description and the analyses of theses regimes. On the one hand, the cathode regime is related to thermal mechanisms as the formation of a vapor phase is obtained at the electrodes’ tip. This vaporization stage appears simultaneously with a transient current measured during the pre-breakdown stage. Two vapor phases are created at each electrode, but the propagation is led by the bush-like structures emerging from the cathode. For a sufficient injected energy (i.e., the electric field above a critical threshold), the vapor phase finally connects both electrodes, leading to the breakdown. On the other hand, the anode regime involves faster processes, which manifest as a thin filament emerging from the anode and propagating toward the cathode, and leading to the breakdown. Schlieren images suggest that the vaporization of the liquid at the cathode does not have time to develop. This observation is corroborated by the absence of transient current before the breakdown. Whatever the regime, the breakdown, corresponding to the voltage drop and a simultaneous current peak, involves a strong emission, the generation of a shock wave, and the formation of a cavitation bubble.

This paper provides additional information on the propagation phase and the breakdown phenomena in order to better understand the processes involved in microsecond discharges in water. These stages seem to be of particular interest regarding the high chemical reactivity required for the different applications. First, the experimental setup is presented; then, the propagation of the discharge for the cathode regime is analyzed using both fast imaging and optical emission spectroscopy; and finally, the bubble pressure resulting from the breakdown is estimated using the Rayleigh–Plesset approach.

## 2. Results and Discussion

### 2.1. Emission during Discharge Propagation of the Cathode Regime

Most of the emission studies reported in the literature have dealt with nonsymmetric configurations, as the most widespread pin-to-plane electrodes, due to the high local electric field at the electrodes’ tip [17,23,24]. As a consequence, the emission analysis was only performed at one electrode. In the frame of this work, the whole region between the two electrodes was considered in order to analyze the initiation and propagation mechanisms. This approach was motivated by the different cathode and anode regimes observed previously by schlieren measurements. 

Due to the limited time resolution of the experimental setup (1.75 µs), the analysis of the anode regime, which is related to fast characteristic time, was not possible. The cathode regime presents a slower propagation evolution that makes its study relevant.

#### 2.1.1. Fast Imaging

**Figure 1:**
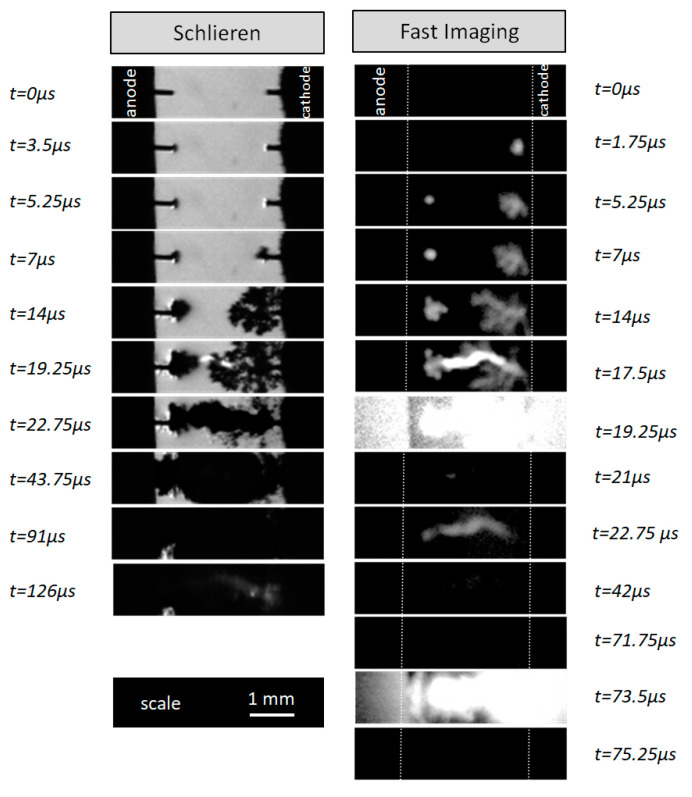
Schlieren imaging and fast imaging obtained for two different experiments representing the cathode regime of electrical breakdown in water using the same initial conditions (U = 12 kV, σ = 100 µS/cm). The corresponding videos are provided as supplementary material.

Schlieren measurements allow the highlighting of the formation of low-density areas, but they do not give information on the chemically active species of the gas during the process. Complementary optical measurements by rapid imaging were carried out in order to visualize the emission all along the discharge propagation. It should be noted that, due to the external light source necessary for the schlieren technique, it is not possible to simultaneously perform schlieren and fast imaging measurements. Figure 1 shows a comparison between schlieren measurements reported previously [20] and the total emission of the discharge for the cathode regime for two different experiments. We observe that the channels propagating from the two electrodes are not only constituted by a gas phase, but that this gas is sufficiently excited to produce a light emission. The pre-breakdown phase consists of ionized gas channels (a plasma has been produced) that propagate from the two electrodes with a bush-like structure. As mentioned previously, the propagation velocity is more important for the channels emerging from the cathode. The connection of the channels leads to the formation of a more intense light channel (also observed in schlieren images) corresponding to the breakdown phenomena. In Figure 1, this breakdown is observed at 19.25 µs on schlieren images (left) and at 17.5 and 19.25 µs on fast imaging (right). 

We mention that for the anode regime (not shown), the initiation of the discharge results in a low-emission area close to the anode, which is immediately followed by a very intense light emission resulting from the breakdown. The time resolution of the experimental setup does not allow observation of the propagation process.

Regarding the secondary breakdowns (t = 126 µs for schlieren images and t = 22.75 µs and t = 73.5 µs for fast imaging in Figure 1), the preceding images do not exhibit emissive zones. The time resolution of the camera is not good enough to visualize the fast propagation of the secondary discharges in the gas phase. In this case, we can only estimate that the propagation speed is greater than 1000 m/s, which is much higher than the propagation speed of the channels observed during the first breakdown (<100 m/s). This is consistent with the assumption that secondary breakdowns initiate and propagate in a gas phase by mechanisms close to streamer propagation. For comparison, the propagation speed of a streamer (in air, at atmospheric pressure) is ~10^6^ m/s. These observations show that the phenomena leading to primary and secondary breakdowns do not take place in the same environment, which is only gaseous for secondary breakdowns, while it involves the liquid phase for primary breakdowns.

**Figure 2:**
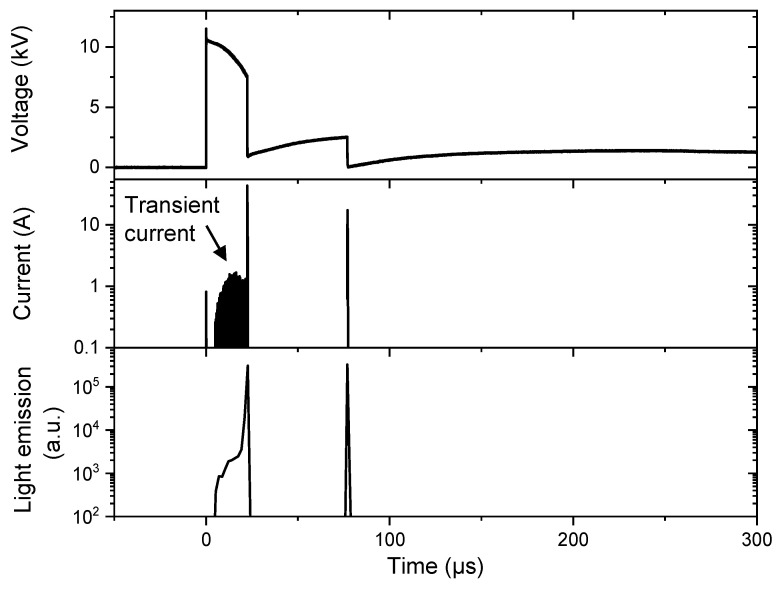
Electrical signals and total emission measured during one single experiment (U = 12 kV, σ = 100 µS/cm, gap = 2 mm).

As mentioned in the introduction, a relationship has been previously highlighted between the time evolution of electrical signals and the gas phase formation for the cathode regime [20]. Similarly, a comparison between the time evolution of the current and the total light emission measured during the discharge was performed. As an example, Figure 2 reports the time evolution of electrical signals and those of the relative intensity of the total light emission. This latter was calculated by adding the relative intensity over all the pixels of the sensor. The comparison of the signals shows that the current peaks occur simultaneously with the high emission peaks. Regarding the light emission, the global intensity is of the same order of magnitude for the two peaks. It should be noted that the fast imaging measurements were not calibrated, because, as shown in Figure 1, pixels can be saturated. As a consequence, the relative intensity of the light emission cannot be rigorously commented on and the time analysis of the discharge was the main concern.

Figure 2 presents the signals related to the experiment depicted by the fast imaging of Figure 1. It shows that the transient current starts simultaneously with the emission signal (at about 5 µs). Considering previous results, showing concomitance between transient current and gas phase creation at the cathode, it is possible to state that the vaporization of the water is accompanied by the excitation of the gas phase. The injected energy is so important that it results in both the water vaporization and the excitation of the species, and also implicitly the chemical activity as dissociation. On the other hand, it is confirmed that neither transient current nor weak light emission is detected before secondary breakdowns.

**Figure 3:**
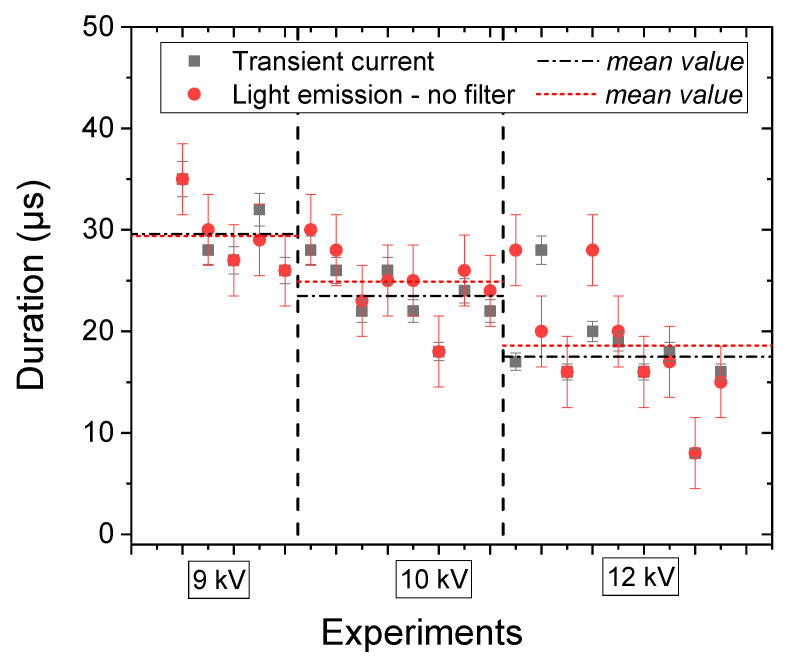
Comparison of duration of the transient current and the total light emission.

While these rapid imaging measurements are qualitative in terms of intensity, they add a number of new insights to the study. For example, we determined the duration of the total emission light during the pre-breakdown. We compared these measurements to the duration of the transient current measured by the oscilloscope, which reflects the formation of the gas channel. Figure 3 presents the results obtained for different applied voltages; each condition has been reproduced several times (from 5 to 9 times) to verify the reproducibility of the results, and the mean value of the durations is represented in dashed lines.

In Figure 3, we observe that the presence of the transient current corresponds to the duration of the total light emission. This result confirms that the formation of the gas phase and the light emission are simultaneous. Moreover, the mean duration of the pre-breakdown phase decreases when the applied voltage increases (from 28 µs for 9 kV to about 18 µs for 12 kV), as the higher electrical field involves a faster propagation of the discharge. As a conclusion, these results highlight the formation and the propagation of a plasma phase from both electrodes. Moreover, they confirm the strong coupling between the applied voltage (electric field) and the physical and chemical mechanisms related to the plasma formation. 

#### 2.1.2. Emission Spectroscopy

In order to better understand the plasma formation processes, optical emission spectroscopy measurements were performed during the pre-breakdown phase of the discharge in water. 

**Figure 4:**
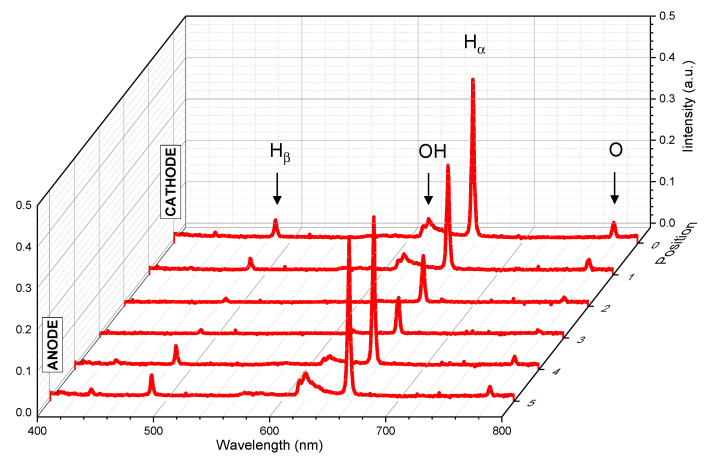
Optical emission spectra (U = 12 kV, σ = 100 µS/cm, gap = 5 mm, spatial resolution of 1 mm, exposure time = 200 µs, 100 accumulations per spectra).

**Figure 5:**
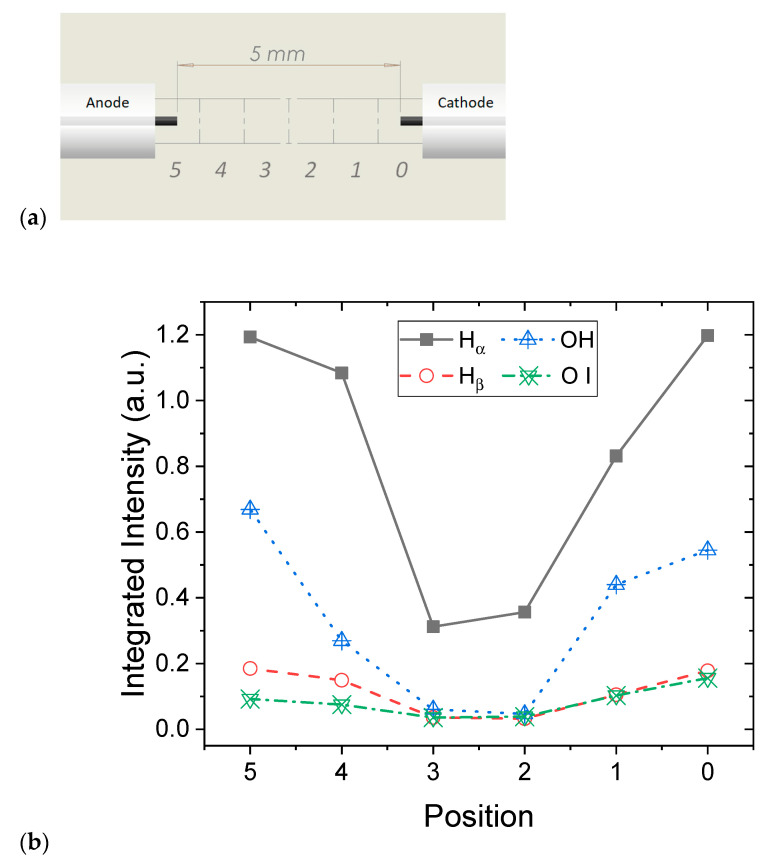
(**a**) Scheme showing the spatial resolution of the OES emission. (**b**) Integrated intensity according to the position between the electrodes for H_α_ (Δλ = 650–666 nm), H_β_ (Δλ = 483–493 nm), OH (Δλ = 610–630 nm), and O (Δλ = 774–783 nm).

The results obtained for the experimental conditions (U = 12 kV, σ = 100 µS/cm) are presented in Figure 4. It should be noted that the weak light emitted during the pre-breakdown phase requires adapted acquisition parameters. The reported emission spectra involve 100 accumulations and the exposure time of one acquisition is 200 µs, which has to be lower than the breakdown duration. To ensure this requirement, some modifications of the experimental conditions have to be carried out. The electrode gap was increased to 5 mm (instead of 2 mm) to enlarge the pre-breakdown duration in order to avoid the emission due to the breakdown that leads to the saturation of the camera. Measurements were performed for different positions every 1 mm in the electrode gap, as depicted in Figure 5a; and position 0 corresponds to the cathode and position 5 is near the anode.

All the monitored spectra shown in Figure 4 report three atomic lines, the hydrogen Balmer lines H_α_ (n = 3→2) and H_β_ (n = 4→2), the oxygen line O I (3p^5^P→ 3s^5^S), and OH molecular bands (second order of A²Σ^+^ X²Π). Obviously, in the water system, the contributions of O, H, and OH were expected as the production of these species results directly from the water dissociation [7]. Most of the studies related to discharges in water have also reported the contribution of these systems [12,17,25,26]. As noted previously, these works presented different experimental conditions as they are related to the nonsymmetric configuration. For the pin-to-pin experiment, we can report that Inoue et al. used the global emission from the whole interelectrode gap focusing on the H_β_ line to estimate the electron density [27].

It should be noted that fast imaging measurements using the interference filter (656 ± 10 nm) were also performed in order to monitor the spatial distribution of the H_α_ (n = 3→2) emission. The imaging of the H_α_ emission shows very similar features to those of the total emission as previously described (Figure 1). An example is provided in the supplementary material (Figure S1). However, the low intensity of the signal is too close to the sensitivity limit of the sensor, so it does not allow a quantitative analysis.

The optical emission spectroscopy diagnostic is known to be very useful to characterize the plasma properties such as the electron and gas temperature. The broadband grating used in this work does not provide the spectral resolution to perform such an analysis, but it gives the spectral overview of all the excited species produced in the gas phase.

Due to the dynamics of the discharge, we observe that the intensity of the emission depends on the position between the two electrodes. Figure 5b shows that, for each position, the intensity of the light emitted by the species is calculated by the integration of the spectra on a given range of wavelengths Δλ (see caption for details). For all the species, the integrated intensity is higher close to the electrodes and lower in the center. Two effects that can be identified in Figure 1 can explain the U-shape observed. On the one hand, close to the electrodes, the emission is local and homogeneous, whereas, away, the discharge evolves with a bush-like structure that may be not entirely collected regarding the spatial resolution of the optical system (1 mm). On the other hand, and more importantly, the emission duration close to the electrode is longer than in the middle considering the whole propagation. Indeed, the emission starts from the electrodes and exists during the entire discharge process, whereas the filaments reach the middle of the gap later. Due to the long exposure time of the acquisitions (200 µs), the variation in the intensity refers to the propagation of the discharge.

An interesting result shown in Figure 5b concerns the relative symmetry of the curves. Considering both the schlieren images and the fast images, we have reported that the discharge propagation of the cathode regime is driven by the propagation of the channels emerging from the cathode. While the dynamics of the discharge is not symmetric, the OES measurements show that the excited species, H_α_, H_β_, OH, and O I, are all present at both electrodes. Moreover, for each species, the local emission (collected using a spatial resolution of 1 mm) presents an intensity of the same order of magnitude at both electrodes. These similarities were not expected, as it is known that the physical mechanisms are different at the cathode and the anode. For the cathode regime, the discharge is initiated by thermal processes, e.g., a vapor bubble is created at the electrodes’ tips by the Joule effect and either field electron emission at the cathode or field ionization at the anode. As a consequence, the mechanisms involving the ionization and the excitation of the species depend on the electrode polarity.

As also observed in Figure 4, the intensity of the H_α_ line is more important than those of the other species on the whole interelectrode gap. Then, close to the electrodes, the intensity of the second order of OH bands is higher than those of H_β_ and O I emission that are relatively similar, whereas, in the middle of the gap, all these three contributions are similar and very weak. This large spectral band overview allows the identification of the species responsible for the emission during the pre-breakdown phase. A closer analysis of these preliminary results has to be performed as the intensity emitted from the species is not sufficient to perform a comparison and to determine plasma characteristics.

The propagation of the discharge leads to the breakdown process, which results in high-intensity emission. Spectra associated with this light emission are difficult to analyze as they present a strong broadening due to several simultaneous effects such as high electron density, pressure, and temperature. As a consequence, the knowledge of the thermodynamic properties of the gas phase produced by the breakdown is necessary to process optical emission spectra.

### 2.2. Bubble Dynamics

**Figure 6:**
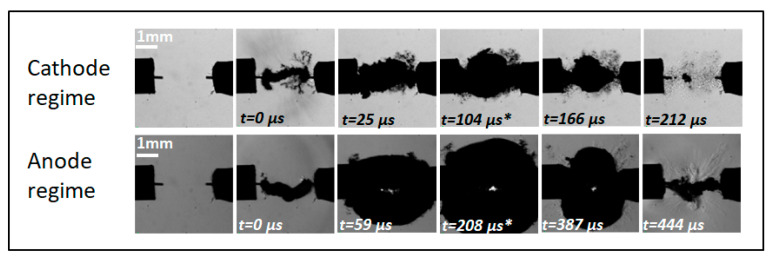
Time evolution of the bubbles obtained after breakdown in cathode and anode regimes (* indicates the maximum radius)—t = 0 μs corresponds to the breakdown (12 kV–100 μS/cm). The corresponding videos are provided as supplementary material.

The measurements of the gas phase characteristics related to the discharges in liquid are not straightforward. This section reports the time evolution of the bubble created by the breakdown [22] and the related pressure. Indeed, the time evolution can be related to the bubble dynamics using the Rayleigh–Plesset model [28,29]. Using this approach, the initial pressure of the bubble for both cathode and anode regimes was determined by simulating the experimental results of bubble radius evolution. It should be noted that as the initiation of the bubble is the breakdown, a new scale was used to describe the bubble dynamics (t = 0 µs is the breakdown), as illustrated in Figure 6.

#### 2.2.1. Modified Rayleigh–Plesset Model

The Rayleigh–Plesset (RP) model is known to be well adapted to simulate spherical bubble dynamics [28,29]. In the frame of this work, because the initial shape of the bubbles is more cylindrical (Figure 6), these models were implemented using an equivalent spherical radius (estimated from the gas phase volume). Moreover, we used a modified RP model in order to take into account the compressibility of both the gas and the liquid [22]:

(1) $$\frac{1}{\rho_{\infty }}\times \left(P_{L}\left(R,t\right)+\frac{R}{C_{l}}\times \frac{d}{dt}P_{g}\left(R\right)-P_{\infty }\right)=R\ddot{R}+\frac{3}{2}{\dot{R}}^{2}$$

where $\rho_{\infty }$ and $P_{\infty }$ are the density and the pressure in the liquid far from the bubble wall, respectively, and $C_{l}$ is the acoustic speed in the liquid. $P_{L}\left(R,t\right)$ is the liquid pressure at the bubble wall defined by:

(2) $$P_{L}\left(R,t\right)=P_{v}\left(T_{\infty }\right)+P_{g}\left(R\right)+\frac{R}{3C_{g}}\times \frac{d}{dt}P_{g}\left(R\right)-\frac{4\mu_{L}\dot{R}}{R}-\frac{2\sigma }{\rho_{\infty }R}$$

where $P_{v}$ is the partial vapor pressure, $\mu_{L}$ is the dynamic viscosity, σ is the surface tension, and $C_{g}$ is the acoustic speed in the gas. $P_{g}\left(R\right)$ is the gas pressure expressed by Equation (3) using a known reference radius $R_{0}$. This latter has been estimated using the theory of equilibrium radius [30]. For the reported experimental conditions (12 kV, 100 µS/cm), a value of about 290 µm is obtained [22].

(3) $$P_{g}\left(R\right)=P_{g_{0}}\times {\left(\frac{R_{0}^{3}-a^{3}}{R^{3}-a^{3}}\right)}^{\gamma }$$

Using the acoustic cavitation approach, it is assumed that the reference pressure $P_{g_{0}}$ in Equation (3) is the equilibrium pressure. This latter can be determined by a simple force balance at the interface of the bubble that results in a value close to the atmospheric pressure. This first approach has been developed previously and gave a satisfying agreement between the time evolution of the experimental radius and the model [22]. However, this model was not able to estimate the values of the pressure, especially the initial pressure, which is largely underestimated.

Another approach is suitable to simulate the bubble dynamics resulting from electrical discharges. It is assumed that the reference pressure $P_{g_{0}}$ is no longer calculated but considered a free parameter for the simulations to fit the experiment. The two parameters that have to be initialized are the initial expansion speed of the bubble $\dot{R}\left(t=0\right)$ and the initial radius R(t=0) (Table 1). $\dot{R}\left(t=0\right)$ was estimated from experimental measurements of the bubble-equivalent spherical radius time evolution (by the tangent method). It is about 15 m/s for the cathode regime and 12.4 m/s for the anode regime Considering the uncertainty of this value, its influence was studied, and it is reported that varying this speed (by a factor of 2 for the cathode regime and up to a factor of 10 for the anode regime) has no significant influence on the simulation results. The initial radius R(t=0) is also very difficult to estimate experimentally. We assumed the initial volume of the gas phase to be cylindrical with a radius of 50 μm (radius of the electrodes), and then we estimated a spherical-equivalent initial radius of R(t=0)=155 µm. In the case of the cathode regime, we also estimated experimentally the initial volume of the gas phase from the first schlieren image after the breakdown (as an example, at 22.75 µs in Figure 1), and then we estimated a spherical-equivalent initial radius of R(t=0)=190 µm. Two simulations were performed in order to analyze the influence of the initial radius.

**Figure 7:**
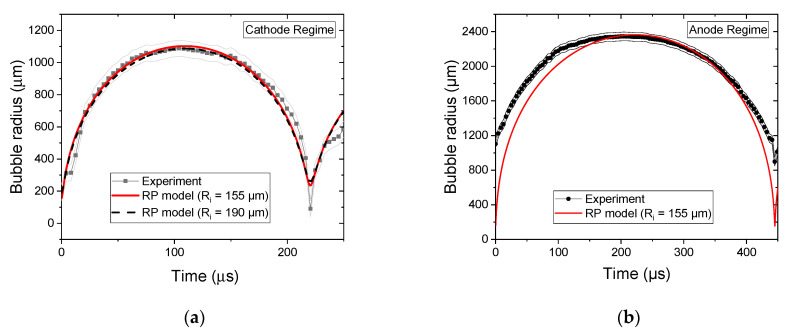
Time evolution of bubble radius. Comparison between experiment and modified Rayleigh–Plesset modeling for (**a**) cathode regime and (**b**) anode regime (grey areas stand for the experimental uncertainty). t = 0 µs corresponds to the breakdown.

The time evolutions of the radius are reported in Figure 7 for the two regimes. The first bubble cycle is very well described for both regimes. We note that the agreement is better for the cathode regime than for the anode regime. Indeed, the anode regime involves a faster propagation of the discharge, which results in a higher uncertainty in the timescale reference (t = 0 µs, which represents the breakdown).

For the cathode regime, Figure 7a shows that the initial radius does not have a major influence on the bubble dynamics, as the variation of 20% of R(t=0) provides very similar results that are both close to the experiment.

#### 2.2.2. Initial Pressure

The bubble pressure is essential to describe and understand the physics and the chemistry resulting from the discharge, but it is hardly attainable experimentally. Indirectly, the initial pressure can be estimated through Rankine–Hugoniot theory knowing the initial propagation speed of the shockwave emitted by the breakdown. In this work, the low time resolution of the diagnostics (1 µs) only allows observation of the pressure wave propagation with a velocity close to the sound speed (1450 m/s), which does not allow estimation of the initial pressure in the bubble.

**Figure 8:**
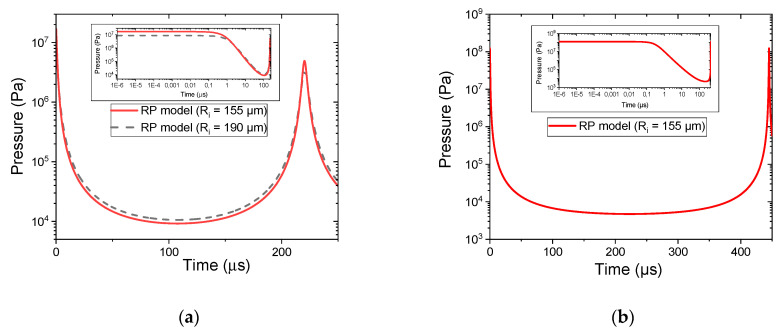
Time evolution of the pressure in the bubble estimated by the modified Rayleigh–Plesset modeling for (**a**) cathode regime and (**b**) anode regime.

The modified Rayleigh–Plesset model applied to bubble dynamics resulting from electrical breakdown in liquid is of great interest as it also provides the time evolution of the pressure inside the bubble. The results obtained for the simulations detailed previously are reported in Figure 8. The energy released during the breakdown (t = 0 µs) leads to increases in the temperature and pressure. The very high pressure in the initial volume of the bubble involves the expansion of the bubble (as presented in Figure 7) and so the decrease in the pressure. The minimum pressure corresponds to the maximum radius of the bubble, when the force balance is equilibrated. Then, the contraction of the bubble involves the increase in the pressure up to a high value.

For the cathode regime (Figure 8a), the initial pressure is estimated to be either 1.7 × 10^7^ Pa or 8.6 × 10^6^ Pa for the initial radius equal to 155 µm or 190 µm, respectively. Due to the adiabatic assumption, the pressure strongly depends on the volume of the initial bubble. The increase of 20% in the initial radius results in a decrease in the initial pressure by a factor of 2 (see inset).

For the anode regime, the initial pressure is about 1.2 × 10^8^ Pa, 7 times higher than that for the cathode regime (for the same initial radius). This higher initial pressure results in a higher maximum radius, in accordance with the adiabatic assumption. The variation observed between the cathode and the anode regimes is consistent with the previous analyses, showing that physical processes of the initiation phase are different. In particular, the difference observed in the initial pressure can be related to different energy injection mechanisms. Indeed, despite the same initial conditions (same total energy), the distribution between the thermal and the mechanical parts of the injected energy is related to the regime: it is about 70/30 for the cathode regime and 50/50 for the anode regime [22].

The values of the initial pressure are consistent with the few that have been reported in the literature for different experimental conditions. For nanosecond discharges, Grosse et al. reported higher values [19]: the analysis of the expansion phase with cavitation theory estimated the initial pressure ranging from 10^9^ to 20 × 10^9^ Pa for a low initial radius (25 µm). For a high energy electrical discharge in water (1.5 kJ), Deroy et al. [15] reported a maximum radius of 48 mm and an initial pressure of about 10^9^ Pa. This high value is due to high storage energy, which also results in a larger bubble. It should be noted that it is not possible to compare the injected energy, as only the stored energy is given (1.5 kJ vs. 100 mJ for this work) and the relationship is not straightforward, as discussed in [22]. Hamdan et al. [31] compared different models to simulate bubble dynamics created by discharges in heptane. They reported values of initial pressure between 1.5 × 10^7^ Pa and 9 × 10^8^ Pa depending on the model and initial parameters of the calculations.

Similarities have also been observed for laser breakdown experiments in water. For example, Vogel et al. [32] reported a value of initial pressure larger than 10^9^ Pa for a bubble formed by a nanosecond pulse, and Lam et al. [33] calculated the initial bubble pressure obtained after laser ablation in water to be equal to 10^7^ Pa.

## 3. Materials and Methods

**Figure 9:**
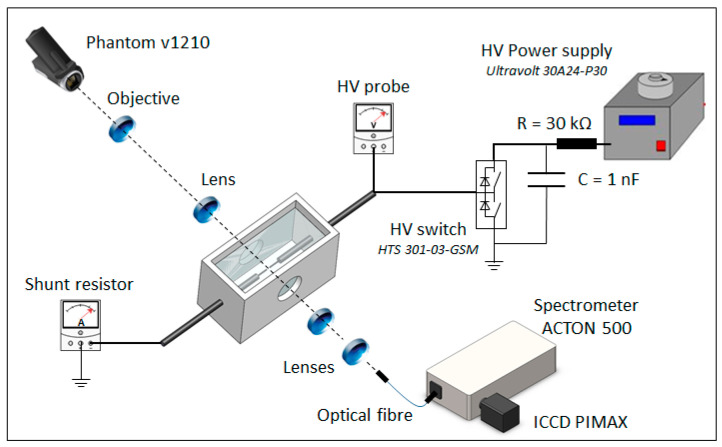
Experimental setup.

The experimental setup is briefly depicted in Figure 9, and more details are provided in [20]. A 150 mL reactor contains two fused silica windows for optical measurements. The discharge is produced through two 100 µm-diameter tungsten electrodes in a horizontal pin-to-pin configuration that are immersed in the liquid. The solution is a mixture of de-ionized water and sodium chloride with a conductivity of 100 µS/cm. The gap distance between the electrodes can vary from 2 to 5 mm using a micrometric controller. The reactor is placed on a micrometric controller that provides the spatial resolution used for OES measurements. 

The pulse generator consists of a 1 nF capacitor constantly charged by a 30 kV high-voltage power supply and discharged using a fast high-voltage solid-state switch (Behlke HTS 301-03-GSM, Behlke Power Electronics GmbH, Kronberg, Germany). It delivers positive high-voltage mono-pulses with a rise time of 30 ns and duration of 500 µs. The applied voltage was changed from 9 to 12 kV. Electrical measurements were carried out using a high-voltage probe (LeCroy PPE20kV, Teledyne Technologies, Chestnut Ridge, NY, USA) and a coaxial current shunt (R = 10 Ω) recorded by a 1 GHz Digital Storage Oscilloscope. These measurements were used to both analyze the time evolution of the discharge and to estimate the injected energy in the discharge [20,22].

The fast imaging was performed using a lenses system focused on a high-speed camera (Phantom V1210, Ametek, Wayne, NJ, USA). Videos were recorded using an exposure time of 0.91 µs with a widescreen resolution of 32 × 128 pixels, giving a time resolution of 1.75 µs. The total light emission from the discharge was monitored.

For the optical emission spectroscopy campaign, a spectrometer (Spectrapro 500i, Teledyne Princeton Instruments, Acton, MA, USA) equipped with an ICCD PIMAX camera (Teledyne Princeton Instruments, Acton, MA, USA) was used. The measurements were carried out using a 50 gr/mm grating providing spectra in the range (400–800) nm with a resolution of 0.51 nm. The light emitted from the plasma was collected by a lenses system and transported via a 1 mm optical fiber core to the entrance slit (20 µm) of the monochromator. This optical setup enables a spatial resolution of 1 mm. The measurements were corrected by the spectral sensitivity of the acquisition devices.

## 4. Conclusions

This paper focused on the experimental study of microsecond electrical discharges in water. New experimental results allow a better understanding of the processes involved during the propagation and the breakdown phases of the discharge.

The time analysis of the total emission intensity showed a relationship between the light emission and the current; especially, during the pre-breakdown phase, the presence of the transient current corresponded to the duration of the total light emission. Plasma was produced during the pre-breakdown phase at both electrodes. Moreover, spatially and spectrally resolved analyses of the emission highlighted the presence of the excited species H, O, and OH in the interelectrode gap. For all species, the emission was as high at both electrodes, and the H_α_ line represented the most intense contribution.

The second analysis concerned the thermodynamic conditions induced by the breakdown of the discharge. The time evolution of the bubble radius was simulated and the estimation of the cavitation bubble pressure was performed using the Rayleigh–Plesset model. Values of about 1.7 × 10^7^ Pa and 1.2 × 10^7^ Pa were reported for the cathode and anode regime, respectively. This multidisciplinary approach constitutes a new step to obtain an accurate physical and chemical description of pin-to-pin electrical discharges in water.

## Figures and Tables

**Table 1:** Model parameters for modified RP simulation.

Regime	R(t = 0)	$\dot{R}\left(t\;=\;0\right)$	P(t = 0)
Cathode	155 µm (190 µm)	15 m/s	170 bar (86 bar)
Anode	155 µm	12.4 m/s	1200 bar

## Data Availability

Data are available from the authors.

## Supplementary Materials

The following are available online, Figure S1: Fast imaging measurements representing the cathode regime of electrical breakdown in water using a 656 ± 10 nm interference filter (U = 12 kV, σ = 100 µS/cm). * indicates that the contrast parameters of the image are different from other images.

## Sample Availability

Samples of the compounds used in this work are available from the authors.
