# Supplementary material for: Microsecond Electrical Breakdown in Water: Advances Using Emission Analysis and Cavitation Bubble Theory
Source: Molecules. 2022 Jan 20;27(3):662. doi: 10.3390/molecules27030662 (PMC8839436; doi:10.3390/molecules27030662)
Supplement: Supplementary file 1 [file molecules-27-00662-s001.zip › Supplementary material.pdf]

Supplementary Material

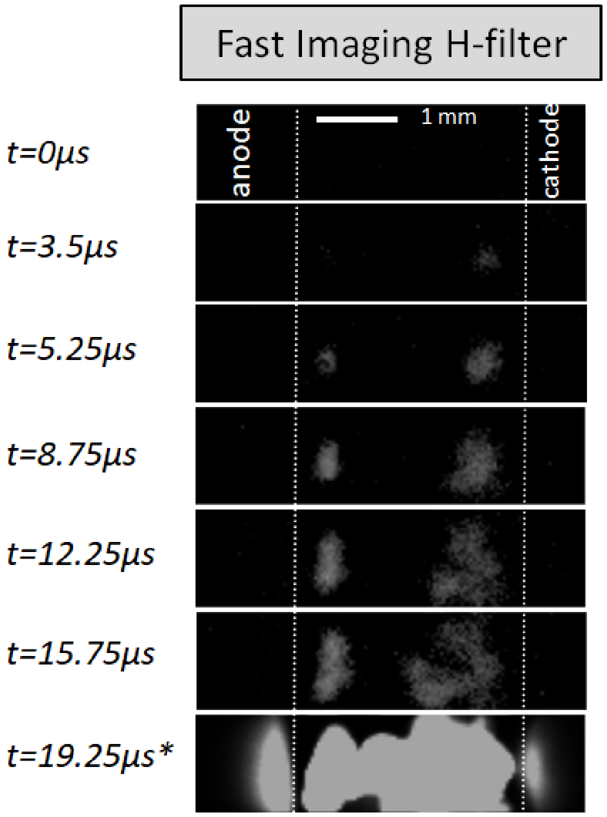

Figure S1. Fast imaging measurements representing the cathode regime of electrical breakdown in water using a 656 ± 10 nm interference filter. (U = 12 kV, σ = 100 µS/cm)—* indicates that the contrast parameters of the image are different from other images.
